# Impact of Fungal Spores on Asthma Prevalence and Hospitalization

**DOI:** 10.3390/ijms23084313

**Published:** 2022-04-13

**Authors:** Kira M. Hughes, Dwan Price, Angel A. J. Torriero, Matthew R. E. Symonds, Cenk Suphioglu

**Affiliations:** 1 NeuroAllergy Research Laboratory (NARL), School of Life and Environmental Sciences, Faculty of Science, Engineering and Built Environment, Deakin University, 221 Burwood Highway, Burwood, VIC 3125, Australia; khughes@deakin.edu.au (K.M.H.); dwan.price@health.vic.gov.au (D.P.); 2 Deakin AIRwatch Pollen and Spore Counting and Forecasting Facility, Deakin University, Burwood, VIC 3125, Australia; 3 Institute for Mental and Physical Health and Clinical Translation (IMPACT), Deakin University, 75 Pigdons Road, Waurn Ponds, VIC 3216, Australia; 4 NeuroAllergy Research Laboratory (NARL), School of Life and Environmental Sciences, Faculty of Science, Engineering and Built Environment, Deakin University, 75 Pigdons Road, Waurn Ponds, VIC 3216, Australia; 5 COVID-19 Response, Department of Health, 50 Lonsdale Street, Melbourne, VIC 3000, Australia; 6 School of Life and Environmental Sciences, Faculty of Science, Engineering and Built Environment, Deakin University, 221 Burwood Highway, Burwood, VIC 3125, Australia; angel.torriero@deakin.edu.au; 7 Centre for Integrative Ecology, School of Life and Environmental Sciences, Faculty of Science, Engineering and Built Environment, Deakin University, 221 Burwood Highway, Burwood, VIC 3125, Australia; matthew.symonds@deakin.edu.au

**Keywords:** asthma, fungal allergy, fungal spore, thunderstorm asthma

## Abstract

Despite making up a significant proportion of airborne allergens, the relationship between fungal spores and asthma is not fully explored. Only 80 taxa of fungi have so far been observed to exacerbate respiratory presentations, with *Cladosporium* spp., *Aspergillus* spp., *Penicillium* spp., and *Alternaria* spp. found to comprise the predominant allergenic airborne spores. Fungal spores have been found in indoor environments, such as hospitals and housing due to poor ventilation. Meanwhile, outdoor fungal spores exhibit greater diversity, and higher abundance and have been associated with hospitalizations from acute asthma presentations. In addition, fungal spores may be the underlying, and perhaps the “missing link”, factor influencing the heightened rate of asthma presentations during epidemic thunderstorm asthma events. To improve our knowledge gap on fungal spores, airborne allergen monitoring must be improved to include not only dominant allergenic fungi but also provide real-time data to accurately and quickly warn the general public. Such data will help prevent future asthma exacerbations and thus save lives. In this review, we examine the health risks of prominent allergenic fungal taxa, the factors influencing spore dispersal and distribution, and why improvements should be made to current sampling methods for public health and wellbeing.

## 1. Introduction

Approximately one in five individuals suffer from allergic rhinitis worldwide, with prevalence increasing in middle-aged years [1]. It is characterized by symptoms presenting after exposure to airborne allergens. Up to 40% of individuals with allergic rhinitis are also diagnosed with asthma, which can bring about aggravated respiratory symptoms following exposure [2]. Under extreme conditions, exposure could provoke acute asthma exacerbations and lead to hospitalization, most notably during thunderstorm asthma events [3]. While making up a large proportion of airborne allergens, fungal spores have until recently been inadequately studied compared to other aeroallergens like pollen [4]. While it is suggested that at least 5 million unique fungal species exist [5], only 80 taxa have been recognized to date regarding their allergenic potential [6]. Airborne spore diversity varies depending on local meteorological and geographical factors but genera commonly attributed to allergies such as *Alternaria* spp., *Aspergillus* spp., *Cladosporium* spp., and *Penicillium* spp. are rampant [7,8]. Atmospheric fungal spore concentrations have been observed exceeding pollen counts by 1000 times or more [9,10], reaching as high as 50,000 spores per m^3^ of air [11]. In addition, fungal spore exposure can last for months as the fungal spore season lasts twice the duration of the pollen season [10], leading to high patient susceptibility for a more significant proportion of the year [12].

## 2. Fungal Spore Morphology

**Figure 1:**
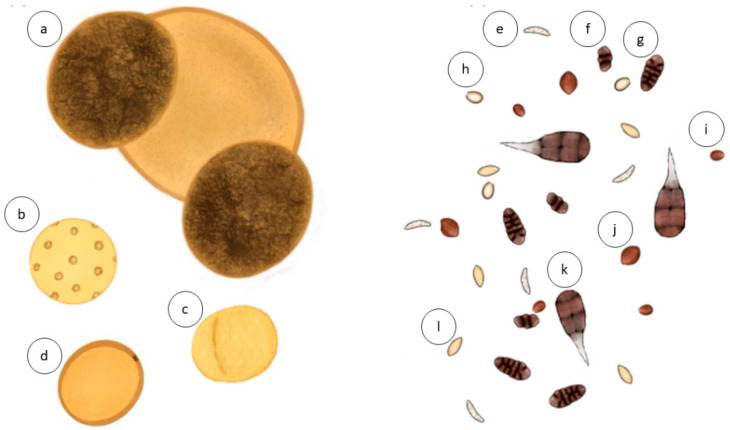
Visualization of the diversity in shape, pigmentation, and ornamentation amongst pollen grains (**left**) and fungal spores (**right**). Pollen grains commonly feature pores, colpi, or reticulated mesh. In contrast, fungal spores have more diverse shapes and ornamented textures. Image features artistic representations of Pine (*Pinaceae*) pollen (**a**), Plantain (*Plantago*) pollen (**b**), Olive (*Oleaceae*) pollen (**c**), Grass (*Poaceae*) pollen (**d**), *Leptosphaeria* spp. spores (**e**), *Pithomyces* spp. spores (**f**), *Pleospora* spp. spores (**g**), *Ganoderma* spp. spores (**h**), *Coprinus* spp. spores (**i**), *Chaetomium* spp. spores (**j**), *Alternaria* spp. spores (**k**), and *Cladosporium* spp. spores (**l**).

Compared to pollen, a high degree of diversity can be observed in the morphology of fungal spores (Figure 1). This diversity can be observed even within the genera level and vary depending on geographical features and environmental conditions [13], such as marine vs. high altitude regions, making it challenging to identify some taxa by observation alone. The ability of spores to disperse and remain viable in the atmosphere is mainly dependent on their morphology. The shape of allergenic fungal spores varies, ranging from allantoid (sausage-shaped) to globular [14,15]. Allantoid fungal spores may have a lower risk of being washed out of the air by wind or rain than rounded spores, allowing them to travel further distances [15]. In addition, the surface of fungal spores can vary from smooth to ornamented textures [14] and, like allantoid-shaped spores, ornamentation provides resistance to getting washed out and aids long-distance dispersal [16]. Furthermore, thick-walled spores are resistant to drying out while pigmented spores are resistant to solar radiation [17]. These adaptations also allow fungal spores to remain in the air for extended periods of time.

Fungal spores usually do not exceed 20 µm in diameter, primarily ranging from 3 to 8 µm [14]. Smaller fungal spores can travel greater distances than larger spores [18]. Due to their small size, most allergenic fungal spores can penetrate deep into the lower airways of sensitized individuals [19], similar to sub-pollen particles from rupturing pollen grains [20]. In addition, fungal spores classed as “thermotolerant” thrive at body temperature. Spores have been found submerged and growing in the aqueous lining of the lower respiratory tract and the lungs’ alveoli [21], which can lead to chronic allergic response and infection [22]. Inhalation and deep penetration of fungal spores can invoke severe allergic or asthmatic symptoms.

## 3. Fungal Sensitization

Fungal sensitization is a potential risk factor for allergic rhinitis and asthma and may contribute to the development of acute respiratory issues [23,24]. The prevalence of fungal allergies is estimated to be 3–10% worldwide [25,26]. Prominent allergenic fungi have been reported to provoke allergic responses in 19–45% of allergic patients and 80% of asthmatic patients from skin tests [27,28,29] and increase the duration of asthmatic symptoms [30]. Atopic workers regularly exposed to fungus and mold spores experience frequent rhinitis and asthma symptoms [31]. About 32.5% of farmers and 16.2% of bakers with occupational asthma are hypersensitive to fungal spores [32].

While Basidiomycota contributes to a majority of aerosol spores, Ascomycota, which make up 4% of airborne fungi, have been strongly linked to allergic rhinitis and asthma [33]. Dominant allergenic fungal taxa, in order of frequency, include *Cladosporium* spp., *Aspergillus* spp., *Penicillium* spp., and *Alternaria* spp. [34,35,36]. These fungal spores have been observed to worsen symptoms in people diagnosed with asthma, especially children [37,38,39,40].

*Alternaria* spp. is one of the most well studied airborne fungi in terms of allergic potency and is one of the few spores recognized by allergy specialists [41]. While *Alternaria* spp. are usually present in lower atmospheric concentrations compared to other allergenic airborne spores, they sport the highest rate of sensitization amongst atopic patients, which is estimated to range from 13–17% [29,42,43], and account for 60% of positive skin prick tests gathered from fungal sensitized patients [8]. Individuals sensitized to *Alternaria* spp. are also likely to be sensitized to one or more other allergenic fungal taxa [43]. This could be explained by cross-reactivity between fungal allergens that share similar proteins [44]. Positive skin tests for *Alternaria* spp. have been associated with the presence of asthma and allergic rhinitis; sensitization was not significantly linked to rhinitis alone [45]. Sensitization from exposure to *Alternaria* spp. usually occurs at a young age [46] and can lead to the development of childhood asthma [47], increasing the frequency of inhaler usage [48,49], and influencing the prevalence of current asthma exacerbations [50]. It is estimated that asthma patients sensitive to *Alternaria* spp. are 20 times more likely to be at risk of respiratory arrest, which can prove fatal [48]. The impact of *Alternaria* spp. on asthma has been observed in Australia, with sensitized people found at risk of airway inflammation and severe respiratory presentations from exposure [51]. In addition, *Alternaria* spp. sensitization has been observed in 13% of hospitalized victims from thunderstorm asthma events in Australia [52]. Immunoglobulin-E specific to *Alternaria* spp. are more prevalent in sensitized young children after exposure [53]. If they suffer from atopic conditions, it could make them highly vulnerable to exposure to allergenic fungal spores.

## 4. Hospitalization from Fungal Spores

Preliminary studies found that asthma-related hospital admissions and deaths increased on days with high fungal spore concentrations [4,54]. It was determined that the concentration of specific allergenic fungi, rather than the overall spore concentration, was strongly associated with causing severe respiratory presentations in sensitized populations (Table 1) [55]. High concentrations of *Alternaria* spp. and *Aspergillus* spp. have been linked to increased hospital admissions for acute asthma exacerbations [56,57] and patients frequently exposed to *Alternaria* spp. were at higher risk of being hospitalized [42].

ICU patients admitted for severe asthma are 20% more likely to be sensitive to at least one fungal allergen than non-ICU patients [58]. For instance, penicillin allergy is reported in 10–20% of hospitalized patients, limiting them to more expensive and less effective antibiotic treatments [59,60]. Patients admitted multiple times to the hospital for asthma are up to 4 times more likely to test positive for fungal sensitivity than patients admitted once or never [61]. In addition, patients admitted multiple times are 10 times more likely to have allergic reactions to more than one fungal allergen [61]. Increased exposure and sensitivity to allergenic fungal spores coincide with the severity of asthma presentations [62,63]. While studies have found that fungal spores could have a more significant impact on hospitalizations than pollen [64,65], further research is required to determine the accuracy of these statements.

**Table 1:** Prevalence and clinical symptoms of prominent allergenic fungal spores.

Fungal Spore	Allergy Prevalence	Environmental Prevalence	Clinical Manifestations	Fatal
*Alternaria* spp.	~13% [66]	Indoors & Outdoors [67]	Allergic Asthma Allergic Rhinitis Allergic Sinusitis [10,44,66,67,68]	Yes (asthma) [69,70]
*Aspergillus* spp.	~2% [66]	Indoors [67]	Allergic Asthma Allergic Bronchopulmonary Mycoses (ABPM) Allergic Rhinitis Hypersensitivity Pneumonitis Mycotoxicosis [44,66,67,71,72]	Yes (lung disease) [73]
*Cladosporium* spp.	~3% [66]	Indoors & Outdoors [67]	Allergic Asthma Allergic Rhinitis Hypersensitivity Pneumonitis [44,66,67,74]	No
*Penicillium* spp.	~2% [66]	Indoors [67]	Allergic Asthma Allergic Rhinitis Mycotoxicosis [44,66,67]	Yes (infection) [75]

## 5. Indoor vs. Outdoor Prevalence

While fungal spores are measured in large quantities outside, fungal spores are also common in the indoor environment, primarily from mold growth due to ventilation systems aiding dispersion. Dominant indoor molds include *Cladosporium* spp., *Aspergillus* spp., and *Penicillium* spp. [76]. Homes analyzed in New York found that 98% contained *Cladosporium* spp. molds and 91% contained *Penicillium* spp. Molds [77]. Approximately one in five hospital departments in Italy reported fungal pollution, with *Aspergillus* spp. making up 91.8% of airborne fungal spore load and 68.5% of molds [78]. Fungal contamination has also been found in neonatal hospital wards [79] and indoor *Penicillium* spp. growth has been identified as the cause of wheezing and breathing problems in newborn infants [80]. Higher rates of allergic reactivity in atopic patients have been reported from exposure to indoor allergens than outdoor allergens [81]. In addition, exposure to indoor fungal spores increases the risk of children developing allergies and asthma over time [82]. However, while the abundance of indoor fungi is dependent on outdoor concentrations and seasonal conditions [83], the level and diversity of indoor fungal spores are exceedingly lower than their counterparts observed outside [84,85]. Furthermore, indoor fungal contamination can be effectively managed via mold removal and remediation [86].

Exposure to *Alternaria* spp. occurs almost always outdoors [10]. Outdoor fungal spores primarily originate from agricultural lands, with variations in climate governing seasonal fluctuations [87]. Sensitization to outdoor fungal spores is more prevalent than sensitization to indoor fungi [88]. Unlike indoor fungi, which have been primarily linked to allergy and infection, fungal spores prevalent outdoors have been reported to cause acute asthma exacerbations [30]. In addition, outdoor spores have been associated with worsening lung function and heightened airway inflammation, especially in asthma patients [89]. Furthermore, an increase in outdoor fungal spore concentrations was linked to a rise in the number of children admitted to hospitals for asthma presentations [90] and deaths caused by asthma [91].

## 6. Climate, Pollution, and Fungal Spores

Approximately half of the variation in airborne fungal spore counts can be explained by changes in weather [92]. High temperatures can increase the rate of spore production and the impact of climate change could see spore production rates continue to rise [93]. High humidity leads to heightened levels of basidiospores but decreases dry-air spores like *Alternaria* spp. and *Cladosporium* spp. [36,40]. Heavy rain has been observed to remove spores from the air, significantly reducing their concentration and the risk of exposure [94].

Temperature, rain, and relative humidity appear to be the more critical factors influencing fungal spore concentration [95]. However, observations of other weather parameters have not produced consistent results. For instance, wind speeds had no noticeable impact on fungal spore concentrations observed in Turkey [96], sampling carried out in Spain found the wind had a negative effect on fungal spore counts [92], while wind speeds observed in Mexico positively influenced spore concentrations [97]. This suggests that other mechanisms must influence fungal spore counts besides climate alone.

Regions with high vehicular and human traffic have been found to contain increased atmospheric concentrations of fungal spores [98]. A high rate of human activities, such as constructions projects, increases environmental disturbances and encourages the dispersal and distribution of airborne fungal spores [99]. In addition, agricultural regions also have significant contributions to spore production. Plant fungal pathogens infect crops to reproduce, with spores continuously distributed via disturbances like wind or farming practices [100].

Urban communities boast higher pollution levels on average, which could also influence spore production and allergenicity. High PM_10_ levels have been associated with increased airborne fungi [27]. Elevated CO_2_ concentrations have been shown to increase *Alternaria* spp. spore production 3-fold [101]. In addition, fungus exposed to heightened CO_2_ levels released spores that contained double the average number of allergenic proteins [101]. Elevated CO_2_ can also lower the resistance of crops to fungal invasion, encouraging the spread and growth of plant fungal pathogens like *Alternaria* spp., which causes early blight [102,103]. However, other pollutants like ozone and NO_2_ have had inconclusive effects on fungal spore levels and need to be explored further [104].

## 7. Fungal Spores and Thunderstorm Asthma

While some studies may have found no consistent links between fungal spore counts and single weather parameters, significant correlations have been found when analyzing variations in weather conditions associated with thunderstorms [105]. Different fungal spore taxa have been observed to increase atmospheric concentrations before, during, and after storms [106]. Elevated levels of *Alternaria* spp. and *Cladosporium* spp. are associated with higher temperatures, high ozone concentration, and low humidity, which is characteristic of conditions prior to a thunderstorm [6,40,107]. Static charges created from lightning strikes can also encourage the release of spores into the air [108]. An increase in airborne fungal spores during storms could be the mechanism that causes severe respiratory exacerbations resulting from epidemic thunderstorm asthma events [109].

Early thunderstorm epidemics were first attributed solely to pollen, particularly ruptured grass pollen grains [110,111,112]. However, the presence of high airborne allergenic spore counts on days with asthma epidemics was identified decades ago [113], and analyzing past epidemics found correlations between spikes in fungal spore levels and the occurrence of thunderstorms [114]. The potential role of fungal spores in epidemic thunderstorm asthma has since been more thoroughly investigated. Fungal spore counts have been observed to double on days with storms as rates of asthma admissions increased [109]. Specifically, high concentrations of *Alternaria spp.* were associated with a rise in asthma presentations across the UK following a thunderstorm, with sensitivity to *Alternaria* spp. increasing an individual’s risk of suffering from thunderstorm-related asthma exacerbations by 900% [115]. High *Cladosporium* spp. levels were also observed to increase emergency department admissions for asthma, which were associated with a higher occurrence of thunderstorms [40]. In addition, ruptured *Alternaria* spp. spores, which are associated with severe asthma presentations, have been collected during thunderstorms [116].

## 8. Airborne Allergen Sampling Methods

**Figure 2:**
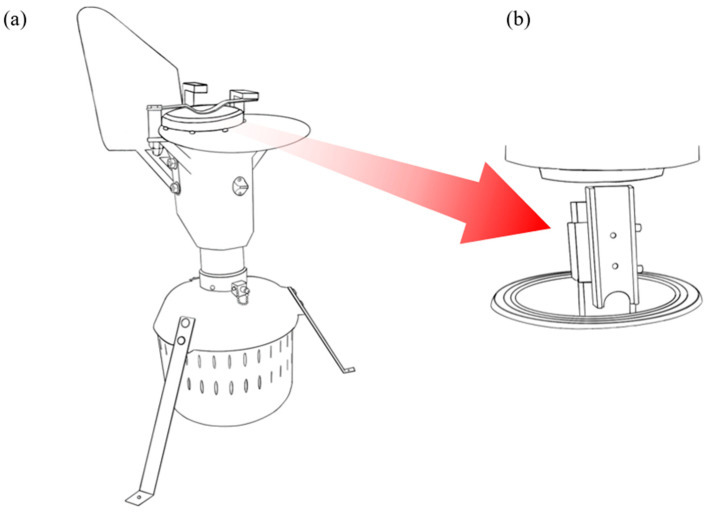
Schematic of Hirst-type 24-h Burkard pollen and spore trap (**a**) and internal microscope slide holder (**b**).

Researchers frequently use sampling traps to collect airborne allergens from internal or external environments. At least 68% of pollen and spore traps currently used worldwide are Hirst-type [117,118]. These sampling traps, first developed in 1952, operate by taking in air volumetrically at 10 L/min and depositing airborne particulates on adhesive-removable surfaces such as tape or microscope slides [119]. The Burkard pollen and spore trap (Figure 2), for instance, is a commonly used Hirst-type sampling device [106] that has been demonstrated to yield high counts of pollen grains and fungal spores and is a reliable method for assessing the atmospheric composition of aeroallergens [120,121].

These traditional sampling traps do have limitations. For instance, sampling machines alone cannot distinguish between local fungal spores and spores dispersed from long distances. Researchers have recently trialed genetically analyzing airborne spores to improve knowledge on local airborne spore diversity, which a sampling trap alone cannot achieve [122]. Similar techniques could be employed at sampling stations across various countries to understand local spore diversity and distribution better. In addition, some Hirst-type sampling traps are less efficient at trapping particulates smaller than 5 µm. While this doesn’t affect larger spores like *Alternaria* spp. and *Cladosporium* spp., it may impact the sampling of smaller allergenic spores like *Aspergillus* spp. and *Penicillium* spp. [123,124]. However, compared to commercially available spore trap services, Hirst-type traps are currently the most appropriate and widely-used method for obtaining fungal spore data [125]. Furthermore, most sampling machines are designed to collect airborne particles over a long period of time, with the Burkard required to be continuously operating for 24 h to 7 days [126]. While this method can provide researchers with a vast amount of uninterrupted data, the delay caused by these long collection times means the public is not provided with real-time information about the current level of airborne allergens. Developing a sampling regime with shorter collection intervals or with the ability to identify pollen or spores automatically would be a significant improvement over traditional sampling traps. Real-time sampling would allow researchers to monitor current atmospheric aeroallergen concentrations in ways that are overlooked or misjudged by Hirst-type traps [127]. Automatic functional pollen counters have been recently found to be effective in regions with low airborne allergen diversity [128], with some models outperforming traditional samplers [127]. However, this technology is still very new and requires further work to fix critical issues such as low accuracy and the high rate of false positives (e.g., non-pollen identified as pollen) [129,130]. With continued investment, automated systems could be adopted for future aeroallergen monitoring.

## 9. Conclusions

Spores pose a significant risk to respiratory health and should be taken more seriously for their allergenic potential. Fungal allergens, both indoors and outdoors, are a common cause of rhinitis and asthma exacerbations and are just as potent as pollen. Limiting the exposure of vulnerable populations to allergenic fungal spores is crucial to preventing severe respiratory exacerbations. Thus, more attention needs to be put towards monitoring seasonal fungal spore concentrations. Updating our daily allergen monitoring systems to include allergenic spores will be necessary to accurately detect airborne allergen levels and help develop warning systems to protect the public during thunderstorm-related asthma epidemics. In addition, more sensitive equipment, potentially with real-time automatic sampling, should be developed to improve current monitoring methods and our ability to collect and identify allergenic fungal spores.

## Data Availability

Not applicable.
